# Crystal structure and characterization of a new copper(II) chloride dimer with meth­yl(pyridin-2-yl­methyl­idene)amine

**DOI:** 10.1107/S2056989020005903

**Published:** 2020-05-05

**Authors:** Olga Yu. Vassilyeva, Elena A. Buvaylo, Vladimir N. Kokozay, Andrii K. Melnyk, Brian W. Skelton

**Affiliations:** a Department of Chemistry, Taras Shevchenko National University of Kyiv, 64/13 Volodymyrska str., Kyiv 01601, Ukraine; b Institute for Sorption and Problems of Endoecology, The National Academy of Sciences of Ukraine, 13 General Naumova str., Kyiv 03164, Ukraine; c School of Molecular Sciences, M310, University of Western Australia, Perth, WA 6009, Australia

**Keywords:** crystal structure, Cu^II^ dimer, Schiff base ligand, 2-pyridine­carbaldehyde, methyl­amine

## Abstract

The new copper(II) complex, namely, di-μ-chlorido-bis­{chlorido­[meth­yl(pyri­din-2-yl­methyl­idene)amine-κ^2^ *N*,*N*′]copper(II)}, [Cu_2_Cl_4_(C_7_H_8_N_2_)_2_], (I), with the ligand 2-pyridyl­methyl-*N*-methyl­imine (*L*, a product of Schiff base condensation between methyl­amine and 2-pyridine­carbaldehyde) is built of discrete centrosymmetric dimers. The coordination about the Cu^II^ ion can be described as distorted square pyramidal. The base of the pyramid consists of two nitro­gen atoms from the bidentate chelate *L* [Cu—N = 2.0241 (9), 2.0374 (8) Å] and two chlorine atoms [Cu—Cl = 2.2500 (3), 2.2835 (3) Å]. The apical position is occupied by another Cl atom with the apical bond being significantly elongated at 2.6112 (3) Å. The *trans* angles of the base are 155.16 (3) and 173.79 (2)°. The Cu**⋯**Cu separation in the dimer is 3.4346 (3) Å. In the crystal structure, the loosely packed dimers are arranged in stacks propagating along the *a* axis. The X-band polycrystalline 77 K EPR spectrum of (I) demonstrates a typical axial pattern characteristic of mononuclear Cu^II^ complexes. Compound (I) is redox active and shows a cyclic voltammetric response with *E*_1/2_ = −0.037 V *versus* silver–silver chloride electrode (SSCE) assignable to the reduction peak of Cu^II^/Cu^I^ in methanol as solvent.

## Abstract 2

The new copper(II) complex [Cu*L*Cl_2_]_2_, where *L* is a product of Schiff base condensation between methyl­amine and 2-pyridine­carbaldehyde, is built of discrete centrosymmetric dimers.

## Chemical context   

The crystal structure of the title compound was determined as part of our ongoing research focused on the design and synthesis of the organic–inorganic halometallates with substituted imidazo[1,5-*a*]pyridinium cations. The first cation in the series, 2-methyl-3-(pyridin-2-yl)imidazo[1,5-*a*]pyridinium, was obtained by the replacement of a conventional aqueous solution of methyl­amine with its solid hydro­chloride salt in the reaction with 2-pyridine­carbaldehyde (2-PCA) in methanol (Buvaylo *et al.*, 2015 ▸). The cation is a result of the acid-catalysed oxidative condensation–cyclization between two mol­ecules of 2-PCA and one mol­ecule of CH_3_NH_2_ with the acid added as an adduct of the amine. The prepared *in situ* organic cation forms a halometallate salt in the subsequent inter­action with divalent metal halides (*M* = Mn, Co, Zn, Cd) or can be isolated in salt form with Cl^−^/NO_3_^−^ anions (Buvaylo *et al.*, 2015 ▸; Vassilyeva *et al.*, 2019*a* ▸,*b* ▸, 2020 ▸).

The burgeoning research in the field of organic–inorganic halometallate-based hybrids in search of new applications (Wheaton *et al.*, 2018 ▸; Yangui *et al.*, 2019 ▸; Szklarz *et al.*, 2020 ▸) prompted us to extend the developed reaction to the possible preparation of a mixed-metal hybrid halometallate by combining two different metals with the organic precursors:

Cu – NiCl_2_·6H_2_O – CH_3_NH_2_·HCl – 2-PCA – CH_3_OH in air

Adhering to the *direct synthesis* approach (Kokozay *et al.*, 2018 ▸), one of the metals was introduced in a zerovalent state. Our earlier studies showed that a metal powder was oxidized in solution to form a coordination compound in the presence of a proton-donating agent and di­oxy­gen from the air, this being reduced to give H_2_O.

Such a complication of the reaction system had an adverse effect, precluding formation of the desired heterocycle with the imidazo[1,5-*a*]pyridinium skeleton but afforded the Schiff base 2-pyridyl­methyl-*N*-methyl­imine (*L*) instead. The latter is a pale-yellow liquid usually accessible by a straightforward inter­action of 2-PCA with a 40% aqueous solution of methyl­amine (Schulz *et al.*, 2009 ▸). In the present work, the imine *L* was isolated as the copper(II) complex [Cu*L*Cl_2_]_2_, (I), the dimeric structure of which has been established by X-ray crystallography. The title compound was characterized by elemental analysis, IR and EPR spectroscopy as well as cyclic voltammetry.
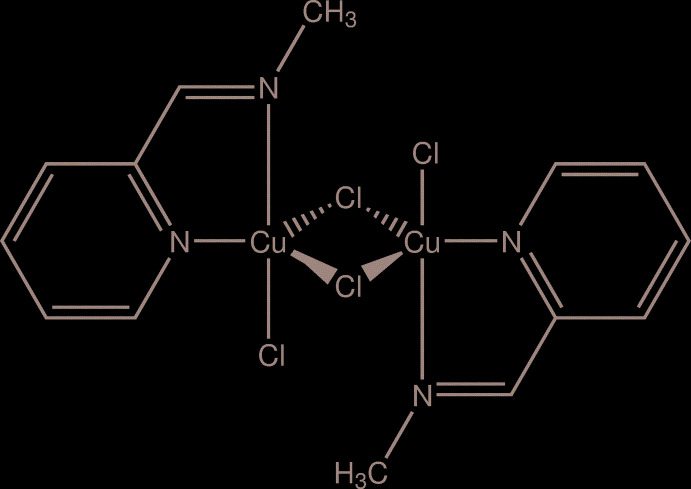


## Structural commentary   

**Figure 1:**
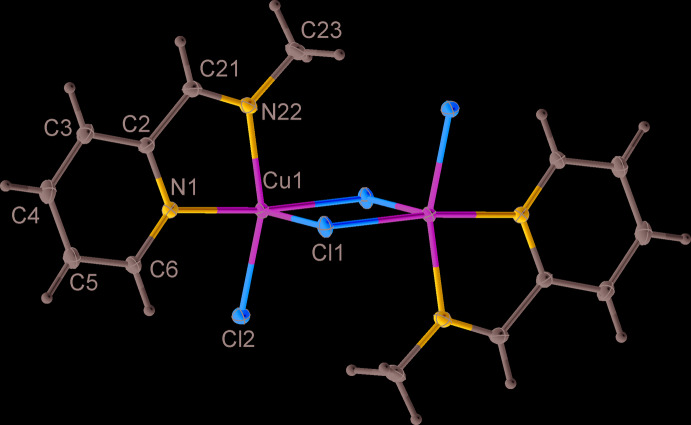
Mol­ecular structure and principal labelling of [Cu*L*Cl_2_]_2_ (I) with ellipsoids at the 50% probability level.

The title complex crystallizes in the triclinic space group, *P* 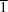; the dimeric mol­ecule is situated on a crystallographic inversion centre. The coordination about the Cu atom can be described as distorted square pyramidal. The angular structural index parameter, τ = (β − α)/60, evaluated from the two largest angles (α < β) in the five-coordinated geometry, which has ideal values of 1 for an equilateral bipyramid and 0 for a square pyramid, is equal to 0.31 (Table 1 ▸). The base of the pyramid consists of the two nitro­gen atoms, N1, N22 from the bidentate chelate ligand *L* and the two chlorine atoms, Cl2 and the centrosymmetrically related Cl1 of the dimer (Fig. 1 ▸). Bond parameters are unexceptional (Table 1 ▸). The apical position is occupied by the Cl1 atom with the apical bond being significantly elongated at 2.6112 (3) Å compared to the Cu1—Cl1^i^ bond length of 2.2835 (3) Å [symmetry code: (i) 1 − *x*, 1 − *y*, 1 − *z*]. The *trans* angles of the base are N22—Cu1—Cl2 = 155.16 (3)° and N1—Cu1—Cl1^i^ = 173.79 (2)°. The *cis* angles at the copper atom vary from 80.20 (3) to 108.803 (10)°. The Cu⋯Cu^i^ separation in the dimer is 3.4346 (3) Å.

## Supra­molecular features   

**Figure 2:**
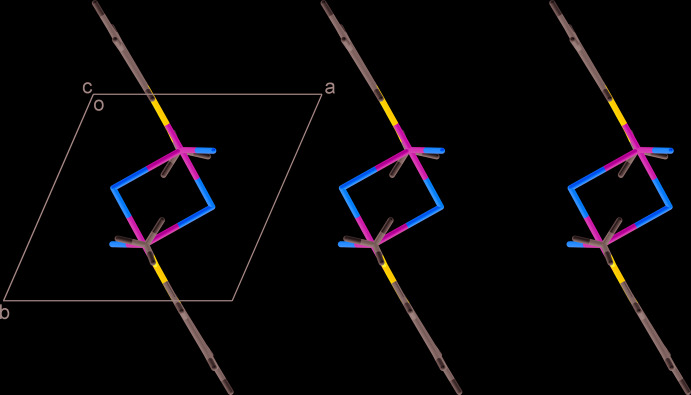
Fragment of crystal packing of [Cu*L*Cl_2_]_2_ (I) viewed along the *c*-axis direction.

In the crystal structure, the dimers are arranged in stacks propagating along the *a-*axis direction and demonstrate loose packing (Fig. 2 ▸). The shortest distance between the Cl atoms of adjacent mol­ecules is 4.4204 (5) Å for Cl2⋯Cl2^ii^ [symmetry code: (ii) 1 − *x*, 2 − *y*, 2 − *z*] and the minimum separation between Cu atoms inside the stack is as long as 7.1550 (5) Å for Cu1⋯Cu1^iii^ [symmetry code: (iii) −*x*, 1 − *y*, 1 − *z*]. The neighbouring pyridyl rings along the stack are coplanar, with the ring centroid distance being equal to the *a*-axis length [7.7054 (5) Å], which is too great for π–overlap.

## Database survey   

A survey of the Cambridge Structural Database (CSD, Version 5.40, October 2019; Groom *et al.*, 2016 ▸) reveals that crystal structures containing *L* as a ligand comprise seven examples of divalent Mn, Ni, Zn and Pd as well as tetra­valent Sn compounds. Among these metal complexes, the ligand demonstrates the same coordination mode as in compound (I) both in monomeric Ni [CSD refcode ADIQOV (Bai *et al.*, 2012 ▸); NEKYOT (Pioquinto-Mendoza *et al.*, 2013 ▸)], Zn (BULSUX; Schulz *et al.*, 2009 ▸), Pd (NEKYUZ; Pioquinto-Mendoza *et al.*, 2013 ▸) and Sn coordination compounds (NELKAS and NELKEW; Guzmán-Percástegui *et al.*, 2013 ▸) and Zn (BULSUX; Schulz *et al.*, 2009 ▸) and Mn (VECDAJ; Bai *et al.*, 2006 ▸) dimers. Out of all the *L* complexes, only the nickel ones accommodate two ligands in the coordination sphere of the metal ion.

## IR and EPR spectroscopy measurements   

A broad band centred at about 3440 cm^−1^ in the IR spectrum of (I) could be due to adsorbed water mol­ecules (see supporting information). Several bands arising above and below 3000 cm^−1^ are assigned to aromatic =CH and alkyl –CH stretching, respectively. The characteristic ν(C=N) absorption of the Schiff base, which appears at 1652 cm^−1^ as a sharp and rather intense band in the IR spectrum of *L* (Schulz *et al.*, 2009 ▸), is detected at 1648 cm^−1^ in the spectrum of (I). A number of sharp and intense absorptions are observed in the aromatic ring stretching (1600–1400 cm^−1^) and C—H out-of-plane bending regions (800–700 cm^−1^).

**Figure 3:**
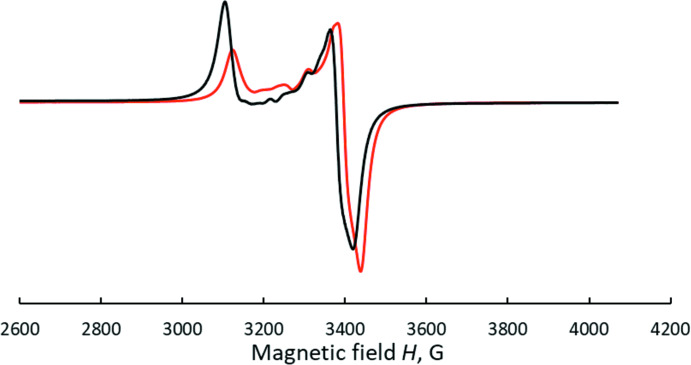
X-band EPR spectra of [Cu*L*Cl_2_]_2_ (I) in the solid state at 293 (red) and 77 K (black).

The X-band polycrystalline EPR spectra of (I) (Fig. 3 ▸) show a typical axial pattern characteristic for the mononuclear Cu^II^ complexes with no visible hyperfine structure. The spectra are almost temperature independent with a subtle change of their shapes seen between 295 and 77 K. The axial symmetry characteristics of (I), *g*_||_ = 2.26 and *g*_⊥_ = 2.06, with a *g*_||_ > *g*_⊥_ > 2.02 relation confirm a square-pyramidal coordination geometry for the metal centre suggested by the structural data. The additional low intensity lines at *g*_eff_ = 2.18, 2.16 and 2.12 may indicate exchange inter­actions between copper(II) ions in the dimer that are probably very weak.

## Cyclic voltammetry   

**Figure 4:**
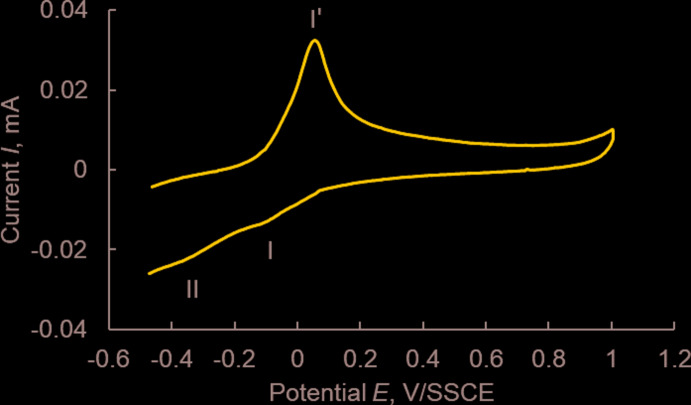
Cyclic voltammogram of [Cu*L*Cl_2_]_2_ (I), 0.1 m*M* in methanol mixed with 0.1 *M* acetate buffer (pH 4) and NaClO_4_ (70:28:2) as supporting electrolyte at a glassy carbon electrode and Ag/AgCl as reference electrode (scan rate: 100 mV s^−1^; *T* = 298 K).

Compound (I) is redox active and shows a cyclic voltammetric response in the potential range of −0.12 – 0.047 V (*E*_1/2_ = −0.037 V *vs* SSCE), which is assignable to the reduction peak of Cu^II^/Cu^I^ (Fig. 4 ▸). The complex exhibits quasi-reversible behaviour as indicated by the non-equivalent current intensity of cathodic and anodic peaks (*i*_c_/*i*_a_ = 0.422) and a large separation between them (167 mV) (Crutchley *et al.*, 1990 ▸). Since Cu^I^ prefers to be four-coordinate, the irreversibility of the Cu^II^/Cu^I^ couple may be due to the dissociation of the dimers in solution. Reduction of copper(I) to copper(0) is associated with the irreversible peak II at −0.36 V *vs* SSCE. The latter process causes removal of the metal centre from the complex mol­ecule. The resulting free ligand undergoes reduction at about −0.8 V, which is superimposed with the reduction peak of the solvent, as is evident from the comparison between the cyclic voltammograms of (I) and supporting electrolyte methanol solutions.

## Synthesis and crystallization   

2-PCA (0.19 ml, 2 mmol) was magnetically stirred with CH_3_NH_2_·HCl (0.27 g, 4 mmol) in 20 ml methanol in a 50 ml Erlenmeyer flask at room temperature (r.t.) for an hour. Dry NiCl_2_·6H_2_O (0.23 g, 1 mmol) and Cu powder (0.06 g, 1.0 mmol) were added to the resulting yellow solution of the preformed Schiff base. The mixture immediately turned green and was magnetically stirred at 323 K in open air to achieve dissolution of the metallic copper (4 h). The resulting solution was filtered and left to evaporate at r.t. Green plate-like crystals of (I) suitable for X-ray analysis deposited over two days. They were filtered off, washed with diethyl ether and finally dried in air. Yield (based on Cu): 71%. Analysis calculated for C_14_H_16_Cl_4_Cu_2_N_4_ (509.19): C 33.02, H 3.17, N 11.00%. Found: C 33.29, H 3.30, N 10.74%. IR (ν, cm^−1^, KBr): 3438*br*, 3092, 3068, 3022, 2992, 2922, 1648, 1598*vs*, 1568, 1474, 1434, 1364, 1300*s*, 1272, 1224, 1156*s*, 1108, 1050, 1024*vs*, 980, 946, 882, 782*vs*, 646, 514, 476, 420.

## Refinement   

Crystal data, data collection and structure refinement details are summarized in Table 2 ▸. Anisotropic displacement parameters were employed for the non-hydrogen atoms. All hydrogen atoms were added at calculated positions and refined by use of a riding model with isotropic displacement parameters based on those of the parent atom (C—H = 0.95 Å, *U*_iso_(H) = 1.2*U*_eq_C for CH, C—H = 0.98 Å, *U*_iso_(H) = 1.5*U*_eq_C for CH_3_).

## Supplementary Material

Crystal structure: contains datablock(s) I, global. DOI: 10.1107/S2056989020005903/pk2625sup1.cif

Structure factors: contains datablock(s) I. DOI: 10.1107/S2056989020005903/pk2625Isup2.hkl

IR spectrum. DOI: 10.1107/S2056989020005903/pk2625sup3.pdf

CCDC reference: 1981381

Additional supporting information:  crystallographic information; 3D view; checkCIF report

## Figures and Tables

**Table 1:** Selected geometric parameters (Å, °)

Cu1—N1	2.0241 (9)	Cu1—Cl1^i^	2.2835 (3)
Cu1—N22	2.0374 (8)	Cu1—Cl1	2.6112 (3)
Cu1—Cl2	2.2500 (3)		
			
N1—Cu1—N22	80.20 (3)	N1—Cu1—Cl1	88.81 (3)
N1—Cu1—Cl2	92.29 (2)	N22—Cu1—Cl1	94.79 (3)
N22—Cu1—Cl2	155.16 (3)	Cl2—Cu1—Cl1	108.803 (10)
N1—Cu1—Cl1^i^	173.79 (2)	Cl1^i^—Cu1—Cl1	91.137 (10)
N22—Cu1—Cl1^i^	93.61 (3)	Cu1^i^—Cl1—Cu1	88.864 (11)
Cl2—Cu1—Cl1^i^	93.600 (11)		

Symmetry code: (i) 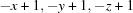.

**Table 2:** Experimental details

Crystal data	
Chemical formula	[Cu_2_Cl_4_(C_7_H_8_N_2_)_2_]
*M*_r_	509.19
Crystal system, space group	Triclinic, *P* 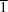
Temperature (K)	100
*a*, *b*, *c* (Å)	7.7054 (5), 7.7240 (5), 8.5606 (5)
α, β, γ (°)	103.659 (5), 98.803 (5), 110.273 (5)
*V* (Å^3^)	448.74 (5)
*Z*	1
Radiation type	Mo *K*α
μ (mm^−1^)	2.97
Crystal size (mm)	0.56 × 0.41 × 0.16

Data collection	
Diffractometer	Oxford Diffraction Gemini diffractometer
Absorption correction	Analytical (*CrysAlis PRO*; Rigaku OD, 2016 ▸)
*T*_min_, *T*_max_	0.367, 0.669
No. of measured, independent and observed [*I* > 2σ(*I*)] reflections	13153, 4238, 3866
*R*_int_	0.024
(sin θ/λ)_max_ (Å^−1^)	0.827

Refinement	
*R*[*F* ^2^ > 2σ(*F* ^2^)], *wR*(*F* ^2^), *S*	0.021, 0.054, 1.08
No. of reflections	4238
No. of parameters	111
H-atom treatment	H-atom parameters constrained
Δρ_max_, Δρ_min_ (e Å^−3^)	0.68, −0.52

Computer programs: *CrysAlis PRO* (Rigaku OD, 2016 ▸), *SHELXT* (Sheldrick, 2015*a* ▸), *SHELXL2017* (Sheldrick, 2015*b* ▸), *DIAMOND* (Brandenburg, 1999 ▸), *Mercury* (Macrae, 2020 ▸) and *WinGX* (Farrugia, 2012 ▸).

## supplementary crystallographic information

### Crystal data

**Table:**

[Cu_2_Cl_4_(C_7_H_8_N_2_)_2_]	*Z* = 1
*M**_r_* = 509.19	*F*(000) = 254
Triclinic, *P*1	*D*_x_ = 1.884 Mg m^−^^3^
Hall symbol: -P 1	Mo *K*α radiation, λ = 0.71073 Å
*a* = 7.7054 (5) Å	Cell parameters from 7922 reflections
*b* = 7.7240 (5) Å	θ = 4.0–37.5°
*c* = 8.5606 (5) Å	µ = 2.97 mm^−^^1^
α = 103.659 (5)°	*T* = 100 K
β = 98.803 (5)°	Plate, green
γ = 110.273 (5)°	0.56 × 0.41 × 0.16 mm
*V* = 448.74 (5) Å^3^	

### Data collection

**Table:**

Oxford Diffraction Gemini diffractometer	4238 independent reflections
Graphite monochromator	3866 reflections with *I* > 2σ(*I*)
Detector resolution: 10.4738 pixels mm^-1^	*R*_int_ = 0.024
ω scans	θ_max_ = 36.0°, θ_min_ = 4.2°
Absorption correction: analytical (CrysAlis Pro; Rigaku OD, 2016)	*h* = −12→12
*T*_min_ = 0.367, *T*_max_ = 0.669	*k* = −12→12
13153 measured reflections	*l* = −14→14

### Refinement

**Table:**

Refinement on *F*^2^	Hydrogen site location: inferred from neighbouring sites
Least-squares matrix: full	H-atom parameters constrained
*R*[*F*^2^ > 2σ(*F*^2^)] = 0.021	*w* = 1/[σ^2^(*F*_o_^2^) + (0.0232*P*)^2^ + 0.1399*P*] where *P* = (*F*_o_^2^ + 2*F*_c_^2^)/3
*wR*(*F*^2^) = 0.054	(Δ/σ)_max_ = 0.002
*S* = 1.08	Δρ_max_ = 0.68 e Å^−^^3^
4238 reflections	Δρ_min_ = −0.52 e Å^−^^3^
111 parameters	Extinction correction: SHELXL-2017/1 (Sheldrick 2015b), Fc^*^=kFc[1+0.001xFc^2^λ^3^/sin(2θ)]^-1/4^
0 restraints	Extinction coefficient: 0.018 (2)

### Special details

**Table:**

Geometry. All esds (except the esd in the dihedral angle between two l.s. planes) are estimated using the full covariance matrix. The cell esds are taken into account individually in the estimation of esds in distances, angles and torsion angles; correlations between esds in cell parameters are only used when they are defined by crystal symmetry. An approximate (isotropic) treatment of cell esds is used for estimating esds involving l.s. planes.
Refinement. One low theta reflection was omitted from the refinement.

### Fractional atomic coordinates and isotropic or equivalent isotropic displacement parameters (Å^2^)

**Table:**

	*x*	*y*	*z*	*U*_iso_*/*U*_eq_	
Cu1	0.50923 (2)	0.72999 (2)	0.57914 (2)	0.01020 (4)	
Cl1	0.73459 (3)	0.54548 (3)	0.59992 (3)	0.01344 (5)	
Cl2	0.36774 (3)	0.72631 (3)	0.79030 (3)	0.01500 (5)	
N1	0.73406 (12)	0.98020 (12)	0.71584 (10)	0.01113 (13)	
C2	0.82713 (14)	1.08198 (14)	0.62321 (12)	0.01197 (15)	
C21	0.74454 (15)	0.99108 (15)	0.44328 (12)	0.01362 (16)	
H21	0.797176	1.050864	0.36667	0.016*	
N22	0.59926 (13)	0.82859 (13)	0.39284 (10)	0.01293 (14)	
C23	0.51841 (17)	0.72996 (17)	0.21469 (12)	0.01786 (18)	
H23A	0.580724	0.81486	0.153155	0.027*	
H23B	0.380672	0.699047	0.18678	0.027*	
H23C	0.539512	0.609696	0.184401	0.027*	
C3	0.98700 (15)	1.25492 (15)	0.69291 (13)	0.01513 (17)	
H3	1.047781	1.324625	0.625083	0.018*	
C4	1.05621 (15)	1.32381 (15)	0.86576 (14)	0.01670 (18)	
H4	1.166445	1.441282	0.917816	0.02*	
C5	0.96252 (15)	1.21913 (15)	0.96081 (13)	0.01547 (17)	
H5	1.008301	1.263236	1.078576	0.019*	
C6	0.79997 (14)	1.04805 (14)	0.88085 (12)	0.01344 (16)	
H6	0.734271	0.977774	0.946118	0.016*	

### Atomic displacement parameters (Å^2^)

**Table:**

	*U*^11^	*U*^22^	*U*^33^	*U*^12^	*U*^13^	*U*^23^
Cu1	0.01109 (6)	0.00923 (6)	0.00822 (5)	0.00253 (4)	0.00183 (4)	0.00182 (4)
Cl1	0.01209 (9)	0.01174 (9)	0.01270 (9)	0.00393 (7)	−0.00076 (7)	0.00086 (7)
Cl2	0.01607 (10)	0.01358 (10)	0.01276 (9)	0.00224 (8)	0.00631 (8)	0.00342 (7)
N1	0.0127 (3)	0.0098 (3)	0.0105 (3)	0.0037 (3)	0.0030 (3)	0.0034 (2)
C2	0.0132 (4)	0.0110 (4)	0.0128 (4)	0.0046 (3)	0.0050 (3)	0.0049 (3)
C21	0.0170 (4)	0.0150 (4)	0.0125 (4)	0.0082 (3)	0.0061 (3)	0.0065 (3)
N22	0.0162 (4)	0.0142 (4)	0.0094 (3)	0.0075 (3)	0.0029 (3)	0.0037 (3)
C23	0.0238 (5)	0.0209 (5)	0.0087 (4)	0.0100 (4)	0.0027 (3)	0.0037 (3)
C3	0.0143 (4)	0.0118 (4)	0.0188 (4)	0.0034 (3)	0.0063 (3)	0.0052 (3)
C4	0.0136 (4)	0.0119 (4)	0.0200 (4)	0.0021 (3)	0.0033 (3)	0.0020 (3)
C5	0.0149 (4)	0.0130 (4)	0.0135 (4)	0.0032 (3)	0.0008 (3)	0.0009 (3)
C6	0.0148 (4)	0.0120 (4)	0.0102 (3)	0.0029 (3)	0.0015 (3)	0.0024 (3)

### Geometric parameters (Å, º)

**Table:**

Cu1—N1	2.0241 (9)	N22—C23	1.4580 (13)
Cu1—N22	2.0374 (8)	C23—H23A	0.98
Cu1—Cl2	2.2500 (3)	C23—H23B	0.98
Cu1—Cl1^i^	2.2835 (3)	C23—H23C	0.98
Cu1—Cl1	2.6112 (3)	C3—C4	1.3953 (15)
N1—C6	1.3320 (12)	C3—H3	0.95
N1—C2	1.3561 (12)	C4—C5	1.3867 (15)
C2—C3	1.3842 (14)	C4—H4	0.95
C2—C21	1.4663 (14)	C5—C6	1.3953 (14)
C21—N22	1.2796 (13)	C5—H5	0.95
C21—H21	0.95	C6—H6	0.95
			
N1—Cu1—N22	80.20 (3)	C21—N22—Cu1	114.21 (7)
N1—Cu1—Cl2	92.29 (2)	C23—N22—Cu1	126.45 (7)
N22—Cu1—Cl2	155.16 (3)	N22—C23—H23A	109.5
N1—Cu1—Cl1^i^	173.79 (2)	N22—C23—H23B	109.5
N22—Cu1—Cl1^i^	93.61 (3)	H23A—C23—H23B	109.5
Cl2—Cu1—Cl1^i^	93.600 (11)	N22—C23—H23C	109.5
N1—Cu1—Cl1	88.81 (3)	H23A—C23—H23C	109.5
N22—Cu1—Cl1	94.79 (3)	H23B—C23—H23C	109.5
Cl2—Cu1—Cl1	108.803 (10)	C2—C3—C4	118.02 (9)
Cl1^i^—Cu1—Cl1	91.137 (10)	C2—C3—H3	121
Cu1^i^—Cl1—Cu1	88.864 (11)	C4—C3—H3	121
C6—N1—C2	118.79 (8)	C5—C4—C3	119.36 (9)
C6—N1—Cu1	127.36 (7)	C5—C4—H4	120.3
C2—N1—Cu1	113.81 (6)	C3—C4—H4	120.3
N1—C2—C3	122.78 (9)	C4—C5—C6	118.99 (10)
N1—C2—C21	113.95 (8)	C4—C5—H5	120.5
C3—C2—C21	123.26 (9)	C6—C5—H5	120.5
N22—C21—C2	117.82 (8)	N1—C6—C5	122.05 (9)
N22—C21—H21	121.1	N1—C6—H6	119
C2—C21—H21	121.1	C5—C6—H6	119
C21—N22—C23	119.31 (9)		
			
C6—N1—C2—C3	−0.69 (15)	N1—C2—C3—C4	1.35 (16)
Cu1—N1—C2—C3	−178.63 (8)	C21—C2—C3—C4	−177.87 (10)
C6—N1—C2—C21	178.59 (9)	C2—C3—C4—C5	−0.67 (16)
Cu1—N1—C2—C21	0.66 (11)	C3—C4—C5—C6	−0.57 (17)
N1—C2—C21—N22	−0.97 (14)	C2—N1—C6—C5	−0.66 (15)
C3—C2—C21—N22	178.31 (10)	Cu1—N1—C6—C5	176.97 (8)
C2—C21—N22—C23	−177.41 (9)	C4—C5—C6—N1	1.29 (17)
C2—C21—N22—Cu1	0.77 (12)		

Symmetry code: (i) −*x*+1, −*y*+1, −*z*+1.

### Hydrogen-bond geometry (Å, º)

**Table:**

*D*—H···*A*	*D*—H	H···*A*	*D*···*A*	*D*—H···*A*
C6—H6···Cl2	0.95	2.71	3.2301 (10)	115
