# Supplementary figures and images for: Crystal structure and characterization of a new copper(II) chloride dimer with meth­yl(pyridin-2-yl­methyl­idene)amine
Source: Acta Crystallogr E Crystallogr Commun. 2020 May 5;76(Pt 6):790–3. doi: 10.1107/S2056989020005903 (PMC7274002; doi:10.1107/S2056989020005903)

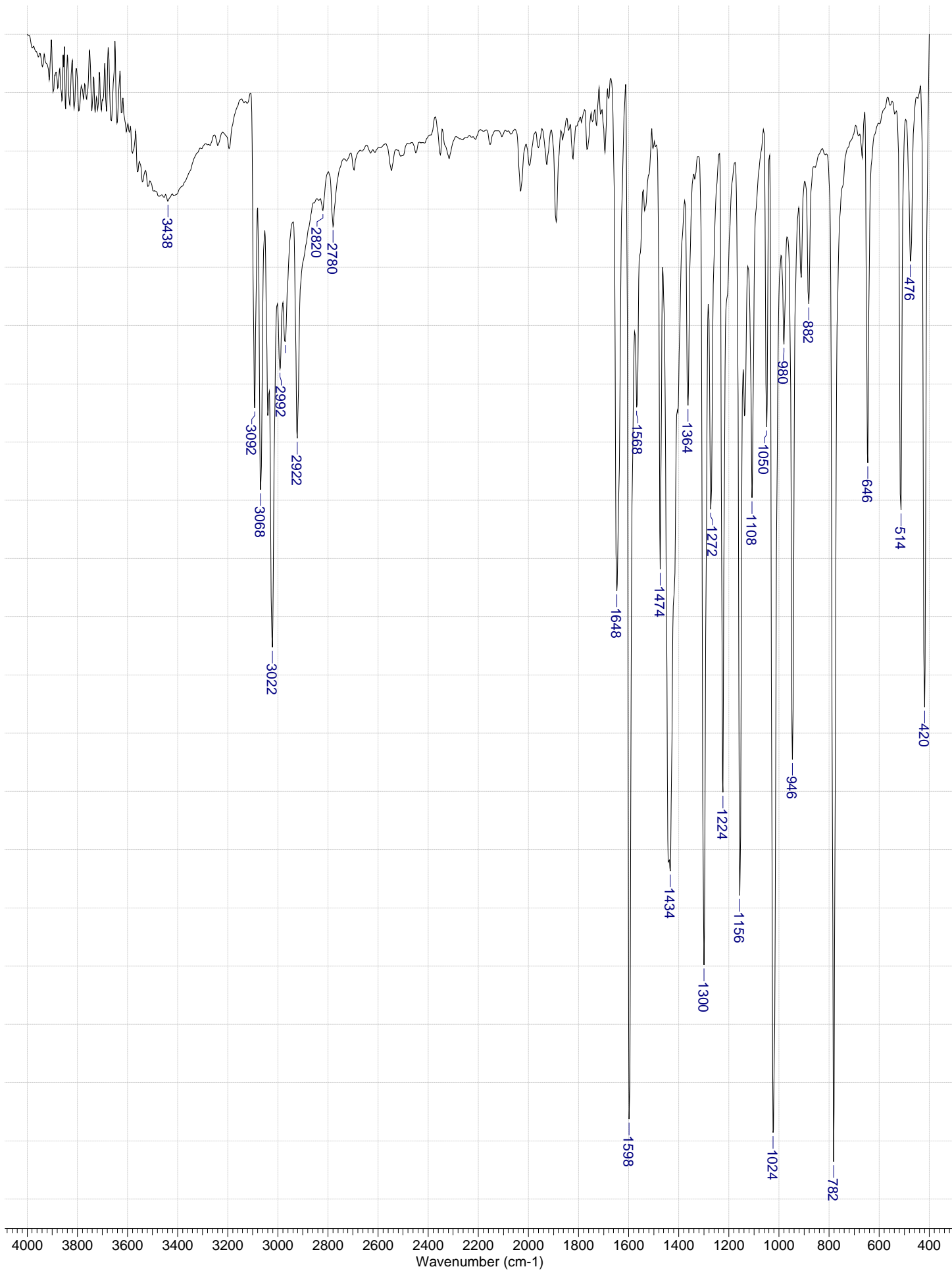

Supplement: Supplementary file 3 [file e-76-00790-sup3.pdf]
